# 

**Published:** 1899-09-23

**Authors:** 


					THE HOSPITAL. ?? The Standard of highest purity."?Lancet. September 23rd, 1899.
CADBURY'S 1* CADBURY'S
COCOA )JL m COCOA
ABSOLUTELY PURE. NO DRUGS OR ALKALIES USED.
?       ^
Ias'coh Western Infirmary
(
No. 678.\jTol. XXVI. [flKSSSJ Satorday?
Sept. 23rd, 1899.
LHaDTeT?*J PRICE TWOPENCE.

				

